# Crystal structure of (3*S*)-3-acet­oxy-17-(pyridin-3-yl)androsta-5,16-diene

**DOI:** 10.1107/S2056989015001966

**Published:** 2015-02-04

**Authors:** Shengjun Zhou, Huaqi Huang, Rongbin Huang

**Affiliations:** a Hangzhou Jiuyuan Gene Engineering Co. Ltd, Hangzhou 310018, Zhejiang, People’s Republic of China; b Department of Chemistry, Xiamen University, Xiamen 361005, People’s Republic of China

**Keywords:** crystal structure, androsta-5,16-diene, hydrogen bonds, *C*(16) chain

## Abstract

In the title compound, C_26_H_33_NO_2_ [systematic name: (3*S*,8*R*,9*S*,10*R*,13*S*,14*S*)-10,13-dimethyl-17-(pyridin-3-yl)-2,3,4,7,8,9,10,11,12,13,14,15-dodeca­hydro-1*H*-cyclo­penta­[*a*]phenanthren-3-yl acetate], the steroid *A*, *B*, *C* and *D* rings adopt chair, half-chair, chair and envelope conformations, respectively. The flap atom of the envelope is the methine C atom fused with the C ring. In the crystal, adjacent mol­ecules, generated by a 2_1_ screw axis, are linked by weak C—H⋯O hydrogen bonds, forming a *C*(16) helical chain running along the *c*-axis direction.

## Related literature   

For inhibition of the androgen signal axis in prostate cancer cells, see: Attard *et al.* (2009 ▸). For use of the title compound as an inhibitor of human cytochrome P450_17*a*_ and the absolute structure of the precursor mol­ecule, see: Potter *et al.* (1995 ▸).
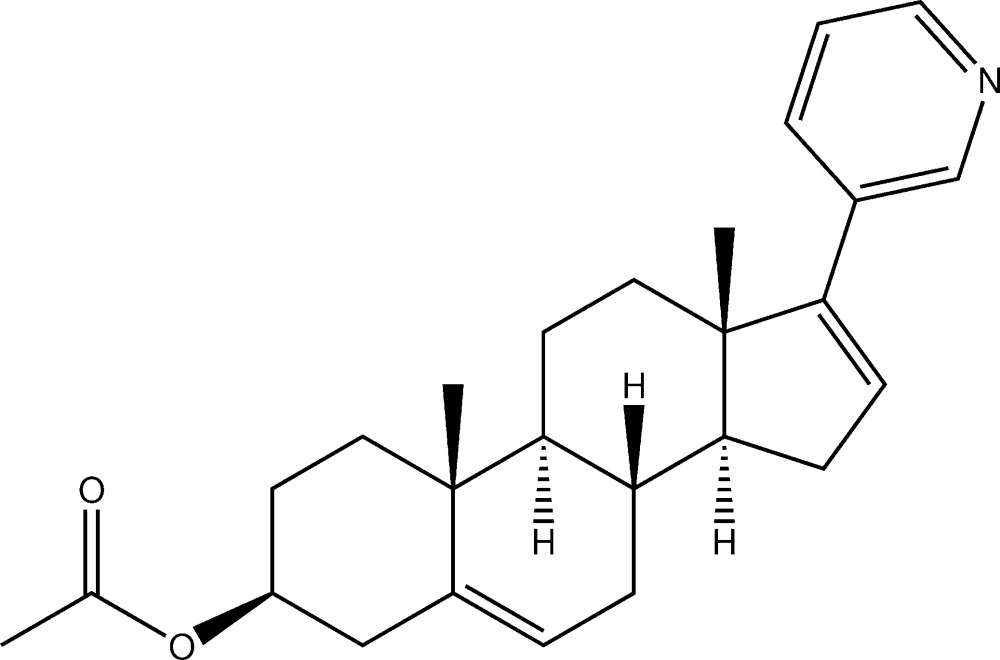


## Experimental   

### Crystal data  

C_26_H_33_NO_2_
*M* *_r_* = 391.53
Orthorhombic, 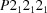

*a* = 7.5180 (5) Å
*b* = 9.7274 (5) Å
*c* = 30.2035 (15) Å
*V* = 2208.8 (2) Å^3^
*Z* = 4
Mo *K*α radiation
μ = 0.07 mm^−1^
*T* = 283 K
0.40 × 0.40 × 0.35 mm

### Data collection  

Bruker SMART APEX 2000 diffractometer
Absorption correction: multi-scan (*SADABS*; Sheldrick, 1996 ▸) *T*_min_ = 0.971, *T*_max_ = 0.975
12750 measured reflections
4320 independent reflections
3261 reflections with *I* > 2σ(*I*)
*R*_int_ = 0.033

### Refinement  

*R*[*F* ^2^ > 2σ(*F* ^2^)] = 0.056
*wR*(*F* ^2^) = 0.128
*S* = 1.06
4320 reflections
262 parameters
H-atom parameters constrained
Δρ_max_ = 0.18 e Å^−3^
Δρ_min_ = −0.20 e Å^−3^

### 

Data collection: *APEX2* (Bruker, 2004 ▸); cell refinement: *SAINT* (Bruker, 2004 ▸); data reduction: *SAINT*; program(s) used to solve structure: *SHELXS97* (Sheldrick, 2008 ▸); program(s) used to refine structure: *SHELXL2014* (Sheldrick, 2015 ▸); molecular graphics: *PLATON* (Spek, 2009 ▸) and *Mercury* (Macrae *et al.*, 2008 ▸); software used to prepare material for publication: *SHELXL2014* and *publCIF* (Westrip, 2010 ▸).

## Supplementary Material

Crystal structure: contains datablock(s) I. DOI: 10.1107/S2056989015001966/hb7357sup1.cif

Structure factors: contains datablock(s) I. DOI: 10.1107/S2056989015001966/hb7357Isup2.hkl

Supporting information file. DOI: 10.1107/S2056989015001966/hb7357Isup3.cml

**Fig. 1.**
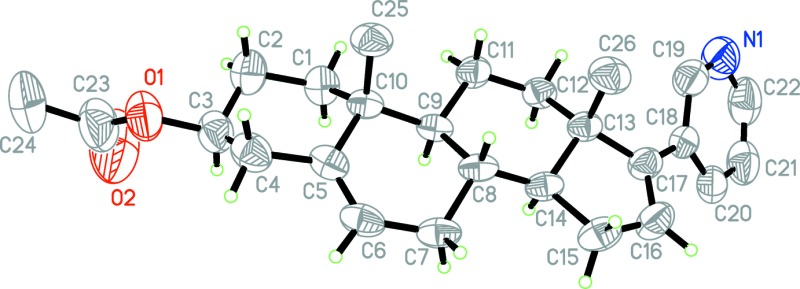
The molecular structure of (I), with displacement ellipsoids are drawn at 50% probability level.

**Fig. 2.**
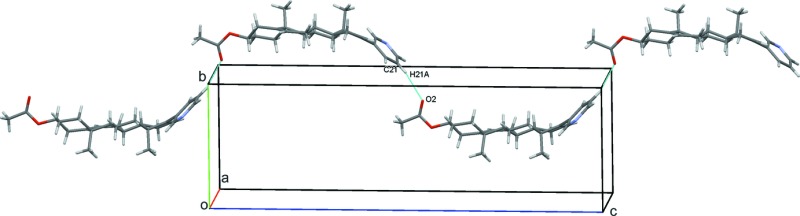
Crystal packing for (I) viewed along the a axis.

. DOI: 10.1107/S2056989015001966/hb7357fig1.tif
The mol­ecular structure of (I), with displacement ellipsoids are drawn at 50% probability level.

a . DOI: 10.1107/S2056989015001966/hb7357fig2.tif
Crystal packing for (I) viewed along the *a* axis.

CCDC reference: 1046309

Additional supporting information:  crystallographic information; 3D view; checkCIF report

## Figures and Tables

**Table 1:** Hydrogen-bond geometry (, )

*D*H*A*	*D*H	H*A*	*D* *A*	*D*H*A*
C21H21*A*O2^i^	0.93	2.70	3.520(6)	147

Symmetry code: (i) 
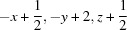
.

## supplementary crystallographic information

### S1. Comment

Castration-resistant prostate cancer (CRPC) is thought to be hormone driven. Inhibition of the androgen signal axis remains an important treatment strategy for CRPC (Attard *et al.*, 2009). The title compound, 3*S*-acetoxy-17-(pyridin-3-yl)androsta-5,16-diene, also referred to as abiraterone acetate, (Scheme I), is a pro-drug for 17-(pyridin-3-yl)androsta-5,16-dien-3P-ol, or abiraterone, a potent inhibitor of human cytochrome P450_17_*_a_* (steroidal 17α-hydroxylase-C~17,20~-lyase). Abiraterone acetate was first synthesized and charaterized by Potter *et al.* (1995), but structural data were not obtained. In this work, we obtained a single-crystal of the title compound (I) and we present its structure here. The space group was non-centrosymmetric confirming the chiral structure. However with no heavy atom, the absolute structure cannot be determined reliably but the absolute structure (Fig.1) has been assigned based on that of the precursor compound dehydro-epiandrosterone (Potter *et al.*, 1995).

In the molecule, there are six chiral carbon atoms. The molecule contains a fused four-ring system. The two saturated six-membered rings adopt chair conformations while the five-membered ring is in an envelope conformation on C14. The six-membered ring with the carbon-carbon double bond approximates to a half chair form. A 2_1_ screw chain running along the *c* axis, Fig. 2 is formed through weak C21–H21A···O2 hydrogen bonds, Table 1.

### S2. Experimental

Abiraterone acetate was synthesized from dehydro-epiandrosterone acetate *via* enol esterification and Suzuki coupling with an overall yield of 72% according to a literature method (Potter, 1995). Colorless blocks were obtained by evaporation from an ethyl acetate solution.

### S3. Refinement

All H atoms bound to carbon were refined using a riding model with d(C—H) = 0.93 Å, for aromatic, 0.98 Å for C—H and 0.97 Å for CH_2_ with *U*_iso_=1.2*U*_eq_ (C). d(C—H) = 0.96 Å with *U*_iso_ = 1.5*U*_eq_ (C) for CH_3_ H atoms. The absolute structure could not be determined reliably and Friedel pairs were merged prior to the final refinement. A Flack parameter is not reported.

### Crystal data

**Table:**

C_26_H_33_NO_2_	*D*_x_ = 1.177 Mg m^−^^3^
*M**_r_* = 391.53	Mo *K*α radiation, λ = 0.71073 Å
Orthorhombic, *P*2_1_2_1_2_1_	Cell parameters from 1526 reflections
*a* = 7.5180 (5) Å	θ = 3.3–26.3°
*b* = 9.7274 (5) Å	µ = 0.07 mm^−^^1^
*c* = 30.2035 (15) Å	*T* = 283 K
*V* = 2208.8 (2) Å^3^	Block, colorless
*Z* = 4	0.40 × 0.40 × 0.35 mm
*F*(000) = 848	

### Data collection

**Table:**

Bruker SMART APEX 2000 diffractometer	4320 independent reflections
Radiation source: Enhance (Mo) X-ray Source	3261 reflections with *I* > 2σ(*I*)
Graphite monochromator	*R*_int_ = 0.033
φ and ω scans	θ_max_ = 26.0°, θ_min_ = 3.4°
Absorption correction: multi-scan (*SADABS*; Sheldrick, 1996)	*h* = −9→9
*T*_min_ = 0.971, *T*_max_ = 0.975	*k* = −11→11
12750 measured reflections	*l* = −37→37

### Refinement

**Table:**

Refinement on *F*^2^	0 restraints
Least-squares matrix: full	Hydrogen site location: inferred from neighbouring sites
*R*[*F*^2^ > 2σ(*F*^2^)] = 0.056	H-atom parameters constrained
*wR*(*F*^2^) = 0.128	*w* = 1/[σ^2^(*F*_o_^2^) + (0.056*P*)^2^ + 0.2757*P*] where *P* = (*F*_o_^2^ + 2*F*_c_^2^)/3
*S* = 1.06	(Δ/σ)_max_ < 0.001
4320 reflections	Δρ_max_ = 0.18 e Å^−^^3^
262 parameters	Δρ_min_ = −0.20 e Å^−^^3^

### Special details

**Table:**

Geometry. All e.s.d.'s (except the e.s.d. in the dihedral angle between two l.s. planes) are estimated using the full covariance matrix. The cell e.s.d.'s are taken into account individually in the estimation of e.s.d.'s in distances, angles and torsion angles; correlations between e.s.d.'s in cell parameters are only used when they are defined by crystal symmetry. An approximate (isotropic) treatment of cell e.s.d.'s is used for estimating e.s.d.'s involving l.s. planes.

### Fractional atomic coordinates and isotropic or equivalent isotropic displacement parameters (Å^2^)

**Table:**

	*x*	*y*	*z*	*U*_iso_*/*U*_eq_	
O1	0.4502 (4)	0.5976 (3)	0.55529 (9)	0.0797 (8)	
O2	0.4415 (9)	0.8126 (5)	0.53343 (14)	0.160 (2)	
N1	−0.1444 (5)	0.7589 (5)	0.93757 (11)	0.0853 (11)	
C1	0.2241 (5)	0.6778 (4)	0.66365 (10)	0.0538 (9)	
H1A	0.0987	0.6823	0.6710	0.065*	
H1B	0.2758	0.7669	0.6701	0.065*	
C2	0.2422 (5)	0.6501 (4)	0.61423 (11)	0.0663 (10)	
H2A	0.1810	0.5653	0.6068	0.080*	
H2B	0.1871	0.7242	0.5977	0.080*	
C3	0.4354 (6)	0.6385 (4)	0.60138 (11)	0.0652 (10)	
H3A	0.4948	0.7270	0.6059	0.078*	
C4	0.5274 (5)	0.5287 (4)	0.62823 (11)	0.0611 (10)	
H4A	0.4796	0.4395	0.6202	0.073*	
H4B	0.6532	0.5288	0.6212	0.073*	
C5	0.5042 (4)	0.5503 (3)	0.67734 (11)	0.0478 (8)	
C6	0.6410 (4)	0.5480 (4)	0.70470 (12)	0.0558 (9)	
H6A	0.7534	0.5393	0.6921	0.067*	
C7	0.6319 (4)	0.5580 (3)	0.75376 (11)	0.0524 (9)	
H7A	0.6799	0.6461	0.7629	0.063*	
H7B	0.7058	0.4866	0.7666	0.063*	
C8	0.4435 (4)	0.5437 (3)	0.77172 (10)	0.0419 (7)	
H8A	0.4106	0.4462	0.7723	0.050*	
C9	0.3129 (4)	0.6220 (3)	0.74174 (10)	0.0383 (7)	
H9A	0.3575	0.7166	0.7404	0.046*	
C10	0.3133 (4)	0.5694 (3)	0.69337 (10)	0.0405 (7)	
C11	0.1258 (4)	0.6319 (4)	0.76150 (10)	0.0489 (8)	
H11A	0.0701	0.5421	0.7597	0.059*	
H11B	0.0560	0.6946	0.7436	0.059*	
C12	0.1198 (4)	0.6809 (3)	0.80970 (10)	0.0459 (8)	
H12A	0.1561	0.7765	0.8111	0.055*	
H12B	−0.0012	0.6745	0.8207	0.055*	
C13	0.2424 (4)	0.5943 (3)	0.83901 (10)	0.0419 (7)	
C14	0.4288 (4)	0.6028 (3)	0.81826 (10)	0.0428 (7)	
H14A	0.4505	0.7015	0.8145	0.051*	
C15	0.5517 (5)	0.5602 (4)	0.85603 (11)	0.0635 (10)	
H15A	0.5603	0.4610	0.8586	0.076*	
H15B	0.6699	0.5989	0.8526	0.076*	
C16	0.4549 (5)	0.6223 (4)	0.89448 (12)	0.0638 (10)	
H16A	0.5077	0.6410	0.9217	0.077*	
C17	0.2853 (4)	0.6475 (3)	0.88538 (10)	0.0496 (8)	
C18	0.1586 (4)	0.7184 (3)	0.91506 (10)	0.0491 (8)	
C19	−0.0234 (5)	0.6956 (4)	0.91332 (12)	0.0684 (11)	
H19A	−0.0637	0.6299	0.8933	0.082*	
C20	0.2145 (6)	0.8159 (4)	0.94512 (11)	0.0621 (10)	
H20A	0.3348	0.8366	0.9477	0.075*	
C21	0.0917 (7)	0.8824 (4)	0.97126 (12)	0.0763 (12)	
H21A	0.1276	0.9469	0.9922	0.092*	
C22	−0.0833 (7)	0.8516 (5)	0.96583 (13)	0.0850 (14)	
H22A	−0.1655	0.8987	0.9831	0.102*	
C23	0.4500 (8)	0.6939 (6)	0.52476 (15)	0.0993 (16)	
C24	0.4541 (9)	0.6347 (7)	0.47920 (15)	0.135 (2)	
H24A	0.4612	0.5363	0.4810	0.202*	
H24B	0.3477	0.6603	0.4637	0.202*	
H24C	0.5559	0.6694	0.4636	0.202*	
C25	0.2137 (5)	0.4319 (3)	0.68890 (12)	0.0564 (9)	
H25A	0.0933	0.4430	0.6989	0.085*	
H25B	0.2135	0.4037	0.6584	0.085*	
H25C	0.2720	0.3633	0.7065	0.085*	
C26	0.1744 (5)	0.4447 (4)	0.84271 (12)	0.0623 (9)	
H26A	0.1471	0.4103	0.8137	0.093*	
H26B	0.2646	0.3884	0.8560	0.093*	
H26C	0.0692	0.4426	0.8607	0.093*	

### Atomic displacement parameters (Å^2^)

**Table:**

	*U*^11^	*U*^22^	*U*^33^	*U*^12^	*U*^13^	*U*^23^
O1	0.107 (2)	0.0719 (17)	0.0607 (15)	−0.0107 (17)	0.0209 (15)	−0.0040 (14)
O2	0.283 (6)	0.092 (3)	0.105 (3)	−0.029 (4)	0.016 (3)	0.029 (2)
N1	0.066 (2)	0.124 (3)	0.066 (2)	0.013 (2)	0.0091 (18)	−0.003 (2)
C1	0.056 (2)	0.051 (2)	0.0536 (19)	0.0134 (17)	0.0029 (16)	0.0016 (15)
C2	0.077 (3)	0.067 (2)	0.055 (2)	0.012 (2)	0.0013 (19)	0.0028 (19)
C3	0.085 (3)	0.056 (2)	0.055 (2)	−0.011 (2)	0.0127 (19)	−0.0096 (17)
C4	0.057 (2)	0.056 (2)	0.071 (2)	0.0000 (18)	0.0142 (18)	−0.0080 (18)
C5	0.0427 (18)	0.0363 (17)	0.064 (2)	0.0015 (14)	0.0108 (16)	−0.0050 (15)
C6	0.0339 (17)	0.053 (2)	0.080 (2)	0.0041 (17)	0.0103 (17)	−0.0085 (18)
C7	0.0323 (16)	0.0496 (19)	0.075 (2)	0.0024 (15)	−0.0032 (16)	−0.0102 (17)
C8	0.0309 (15)	0.0328 (15)	0.0620 (19)	0.0017 (14)	−0.0061 (14)	−0.0040 (14)
C9	0.0295 (14)	0.0295 (15)	0.0558 (18)	0.0009 (12)	−0.0011 (13)	−0.0013 (13)
C10	0.0351 (15)	0.0308 (15)	0.0556 (18)	−0.0008 (13)	−0.0011 (13)	−0.0010 (14)
C11	0.0363 (17)	0.059 (2)	0.0512 (18)	0.0081 (16)	−0.0038 (14)	0.0012 (16)
C12	0.0338 (16)	0.0510 (18)	0.0528 (18)	0.0071 (14)	−0.0013 (14)	0.0011 (15)
C13	0.0357 (15)	0.0380 (17)	0.0520 (17)	0.0016 (14)	−0.0053 (13)	0.0023 (14)
C14	0.0337 (15)	0.0364 (15)	0.0583 (18)	0.0038 (14)	−0.0088 (14)	−0.0007 (14)
C15	0.0433 (18)	0.074 (2)	0.073 (2)	0.0098 (19)	−0.0169 (18)	−0.002 (2)
C16	0.058 (2)	0.078 (3)	0.056 (2)	0.007 (2)	−0.0171 (18)	−0.0013 (18)
C17	0.0467 (19)	0.049 (2)	0.0528 (18)	−0.0002 (16)	−0.0094 (15)	0.0066 (15)
C18	0.049 (2)	0.056 (2)	0.0423 (16)	0.0006 (17)	−0.0048 (15)	0.0081 (16)
C19	0.061 (2)	0.090 (3)	0.054 (2)	0.000 (2)	−0.0017 (18)	−0.005 (2)
C20	0.076 (3)	0.066 (2)	0.0447 (17)	−0.010 (2)	0.0002 (18)	0.0053 (18)
C21	0.102 (4)	0.076 (3)	0.051 (2)	−0.003 (3)	0.009 (2)	−0.0027 (19)
C22	0.098 (4)	0.103 (4)	0.054 (2)	0.025 (3)	0.014 (2)	0.005 (2)
C23	0.127 (4)	0.099 (4)	0.072 (3)	−0.019 (4)	0.022 (3)	0.016 (3)
C24	0.162 (5)	0.178 (6)	0.064 (3)	−0.021 (5)	0.037 (3)	0.008 (3)
C25	0.057 (2)	0.0446 (19)	0.068 (2)	−0.0136 (17)	0.0004 (17)	−0.0082 (17)
C26	0.062 (2)	0.047 (2)	0.077 (2)	−0.0094 (19)	−0.0028 (19)	0.0058 (18)

### Geometric parameters (Å, º)

**Table:**

O1—C23	1.314 (5)	C11—H11B	0.9700
O1—C3	1.452 (4)	C12—C13	1.531 (4)
O2—C23	1.185 (6)	C12—H12A	0.9700
N1—C19	1.321 (5)	C12—H12B	0.9700
N1—C22	1.324 (6)	C13—C17	1.527 (4)
C1—C2	1.523 (4)	C13—C14	1.537 (4)
C1—C10	1.538 (4)	C13—C26	1.546 (4)
C1—H1A	0.9700	C14—C15	1.526 (4)
C1—H1B	0.9700	C14—H14A	0.9800
C2—C3	1.507 (6)	C15—C16	1.498 (5)
C2—H2A	0.9700	C15—H15A	0.9700
C2—H2B	0.9700	C15—H15B	0.9700
C3—C4	1.509 (5)	C16—C17	1.328 (5)
C3—H3A	0.9800	C16—H16A	0.9300
C4—C5	1.508 (4)	C17—C18	1.479 (5)
C4—H4A	0.9700	C18—C20	1.378 (5)
C4—H4B	0.9700	C18—C19	1.387 (5)
C5—C6	1.320 (5)	C19—H19A	0.9300
C5—C10	1.526 (4)	C20—C21	1.376 (5)
C6—C7	1.487 (4)	C20—H20A	0.9300
C6—H6A	0.9300	C21—C22	1.359 (7)
C7—C8	1.524 (4)	C21—H21A	0.9300
C7—H7A	0.9700	C22—H22A	0.9300
C7—H7B	0.9700	C23—C24	1.492 (7)
C8—C14	1.523 (4)	C24—H24A	0.9600
C8—C9	1.537 (4)	C24—H24B	0.9600
C8—H8A	0.9800	C24—H24C	0.9600
C9—C11	1.531 (4)	C25—H25A	0.9600
C9—C10	1.548 (4)	C25—H25B	0.9600
C9—H9A	0.9800	C25—H25C	0.9600
C10—C25	1.538 (4)	C26—H26A	0.9600
C11—C12	1.532 (4)	C26—H26B	0.9600
C11—H11A	0.9700	C26—H26C	0.9600
			
C23—O1—C3	118.5 (3)	C11—C12—H12A	109.4
C19—N1—C22	115.8 (4)	C13—C12—H12B	109.4
C2—C1—C10	114.3 (3)	C11—C12—H12B	109.4
C2—C1—H1A	108.7	H12A—C12—H12B	108.0
C10—C1—H1A	108.7	C17—C13—C12	118.1 (3)
C2—C1—H1B	108.7	C17—C13—C14	99.4 (2)
C10—C1—H1B	108.7	C12—C13—C14	106.5 (2)
H1A—C1—H1B	107.6	C17—C13—C26	108.8 (3)
C3—C2—C1	110.6 (3)	C12—C13—C26	111.1 (3)
C3—C2—H2A	109.5	C14—C13—C26	112.4 (3)
C1—C2—H2A	109.5	C8—C14—C15	123.0 (3)
C3—C2—H2B	109.5	C8—C14—C13	115.0 (2)
C1—C2—H2B	109.5	C15—C14—C13	103.4 (3)
H2A—C2—H2B	108.1	C8—C14—H14A	104.6
O1—C3—C2	109.9 (3)	C15—C14—H14A	104.6
O1—C3—C4	106.6 (3)	C13—C14—H14A	104.6
C2—C3—C4	110.9 (3)	C16—C15—C14	100.1 (3)
O1—C3—H3A	109.8	C16—C15—H15A	111.7
C2—C3—H3A	109.8	C14—C15—H15A	111.7
C4—C3—H3A	109.8	C16—C15—H15B	111.7
C5—C4—C3	112.1 (3)	C14—C15—H15B	111.7
C5—C4—H4A	109.2	H15A—C15—H15B	109.5
C3—C4—H4A	109.2	C17—C16—C15	112.4 (3)
C5—C4—H4B	109.2	C17—C16—H16A	123.8
C3—C4—H4B	109.2	C15—C16—H16A	123.8
H4A—C4—H4B	107.9	C16—C17—C18	125.4 (3)
C6—C5—C4	121.6 (3)	C16—C17—C13	109.2 (3)
C6—C5—C10	122.4 (3)	C18—C17—C13	125.3 (3)
C4—C5—C10	115.9 (3)	C20—C18—C19	115.9 (3)
C5—C6—C7	126.0 (3)	C20—C18—C17	121.6 (3)
C5—C6—H6A	117.0	C19—C18—C17	122.5 (3)
C7—C6—H6A	117.0	N1—C19—C18	125.7 (4)
C6—C7—C8	113.1 (3)	N1—C19—H19A	117.1
C6—C7—H7A	109.0	C18—C19—H19A	117.1
C8—C7—H7A	109.0	C21—C20—C18	119.8 (4)
C6—C7—H7B	109.0	C21—C20—H20A	120.1
C8—C7—H7B	109.0	C18—C20—H20A	120.1
H7A—C7—H7B	107.8	C22—C21—C20	118.4 (4)
C14—C8—C7	111.2 (2)	C22—C21—H21A	120.8
C14—C8—C9	108.1 (2)	C20—C21—H21A	120.8
C7—C8—C9	109.8 (3)	N1—C22—C21	124.3 (4)
C14—C8—H8A	109.3	N1—C22—H22A	117.8
C7—C8—H8A	109.3	C21—C22—H22A	117.8
C9—C8—H8A	109.3	O2—C23—O1	122.6 (5)
C11—C9—C8	112.8 (2)	O2—C23—C24	125.5 (5)
C11—C9—C10	113.0 (2)	O1—C23—C24	111.8 (5)
C8—C9—C10	113.0 (2)	C23—C24—H24A	109.5
C11—C9—H9A	105.7	C23—C24—H24B	109.5
C8—C9—H9A	105.7	H24A—C24—H24B	109.5
C10—C9—H9A	105.7	C23—C24—H24C	109.5
C5—C10—C25	108.9 (3)	H24A—C24—H24C	109.5
C5—C10—C1	107.9 (3)	H24B—C24—H24C	109.5
C25—C10—C1	109.4 (3)	C10—C25—H25A	109.5
C5—C10—C9	109.9 (2)	C10—C25—H25B	109.5
C25—C10—C9	111.7 (3)	H25A—C25—H25B	109.5
C1—C10—C9	108.8 (2)	C10—C25—H25C	109.5
C9—C11—C12	114.6 (2)	H25A—C25—H25C	109.5
C9—C11—H11A	108.6	H25B—C25—H25C	109.5
C12—C11—H11A	108.6	C13—C26—H26A	109.5
C9—C11—H11B	108.6	C13—C26—H26B	109.5
C12—C11—H11B	108.6	H26A—C26—H26B	109.5
H11A—C11—H11B	107.6	C13—C26—H26C	109.5
C13—C12—C11	111.1 (2)	H26A—C26—H26C	109.5
C13—C12—H12A	109.4	H26B—C26—H26C	109.5

### Hydrogen-bond geometry (Å, º)

**Table:**

*D*—H···*A*	*D*—H	H···*A*	*D*···*A*	*D*—H···*A*
C21—H21*A*···O2^i^	0.93	2.70	3.520 (6)	147

Symmetry code: (i) −*x*+1/2, −*y*+2, *z*+1/2.
