# Supplementary figures and images for: Crystal structure of (3S)-3-acet­oxy-17-(pyridin-3-yl)androsta-5,16-diene
Source: Acta Crystallogr E Crystallogr Commun. 2015 Feb 4;71(Pt 3):o146–7. doi: 10.1107/S2056989015001966 (PMC4350699; doi:10.1107/S2056989015001966)

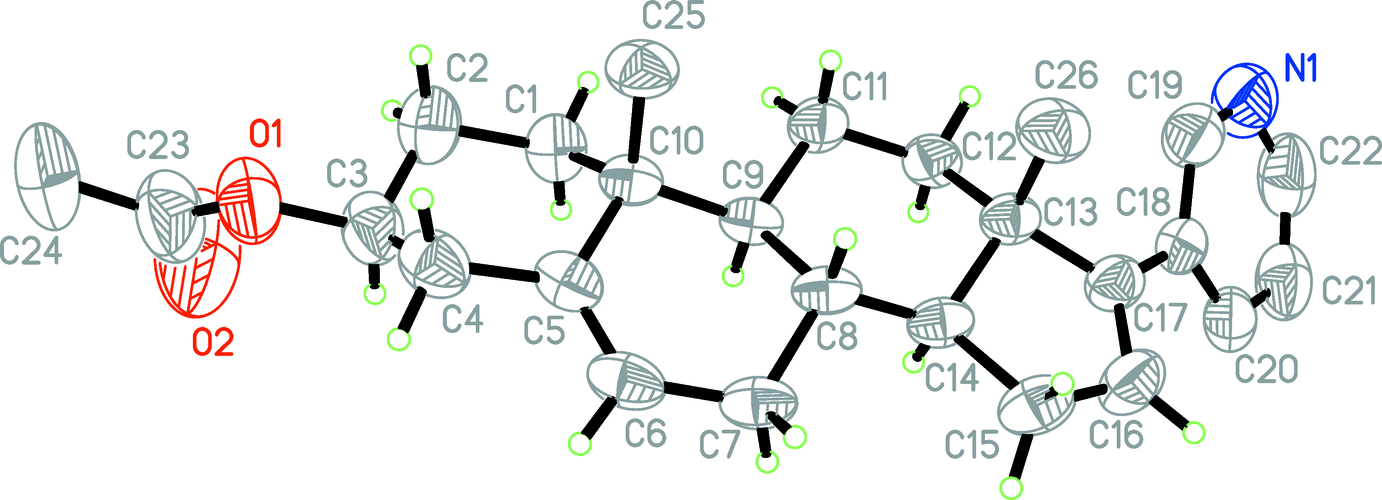

Supplement: Supplementary file 4 [file e-71-0o146-fig1.tif]

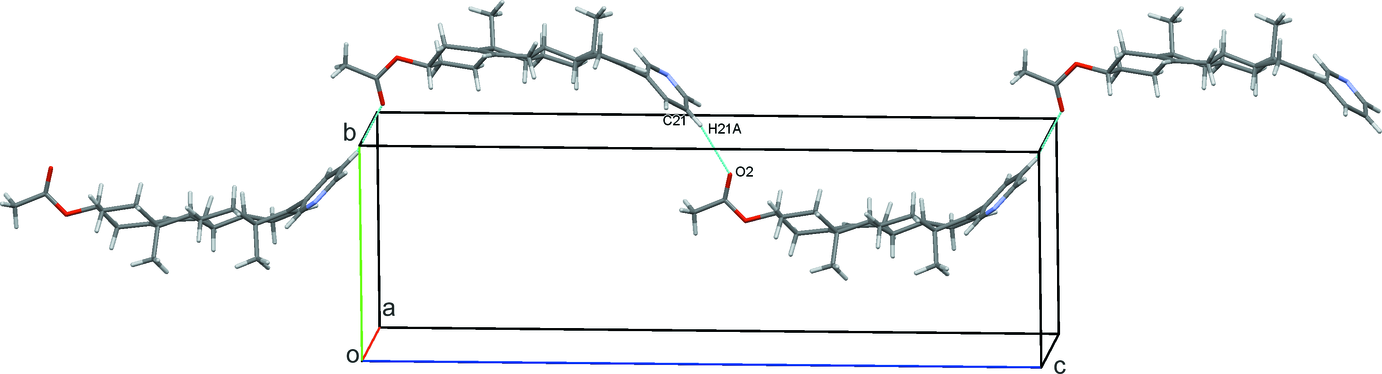

Supplement: Supplementary file 5 [file e-71-0o146-fig2.tif]
